# Incidence and Nature of Short-Term Adverse Events following COVID-19 Second Boosters: Insights from Taiwan’s Universal Vaccination Strategy

**DOI:** 10.3390/vaccines12020149

**Published:** 2024-01-31

**Authors:** Ching-Hao Lin, Tsung-An Chen, Pin-Hsuan Chiang, Ai-Ru Hsieh, Bih-Ju Wu, Po-Yu Chen, Kuan-Chen Lin, Zih-Syun Tsai, Ming-Hwai Lin, Tzeng-Ji Chen, Yu-Chun Chen

**Affiliations:** 1 Department of Family Medicine, Taipei Veterans General Hospital, Taipei 112, Taiwan; celvanse@gmail.com (C.-H.L.); firen8056@gmail.com (T.-A.C.); zzzlccfyp@gmail.com (K.-C.L.); mhlin@vghtpe.gov.tw (M.-H.L.); tjchen@vhct.gov.tw (T.-J.C.); 2 Big Data Center, Taipei Veterans General Hospital, Taipei 112, Taiwan; s94120506@gmail.com (P.-H.C.); zihsyun.k@gmail.com (Z.-S.T.); 3 Department of Statistics, Tamkang University, New Taipei City 251, Taiwan; airudropbox@gmail.com; 4 Department of Nursing, Taipei Veterans General Hospital, Taipei 112, Taiwan; bjwu@vghtpe.gov.tw; 5 Department of Family Medicine, Cheng Hsin General Hospital, Taipei 112, Taiwan; barry50710@gmail.com; 6 School of Medicine, National Yang Ming Chiao Tung University, Taipei 112, Taiwan; 7 Department of Family Medicine, Taipei Veterans General Hospital, Hsinchu Branch, Hsinchu 31064, Taiwan

**Keywords:** COVID-19 booster vaccination, adverse events, vaccine safety, immunization strategies, pharmacovigilance, mRNA vaccines, Taiwan’s vaccination program, real-world evidence

## Abstract

This study evaluates the incidence and characteristics of adverse events (AEs) following the second COVID-19 booster dose, leveraging Taiwan’s distinctive approach of extending booster vaccinations to all citizens, unlike the targeted high-risk group strategies in other countries. Utilizing data from Taipei Veterans General Hospital’s Vaccine Adverse Event Reporting System (VAERS) from 27 October 2022 to 19 January 2023, this research examines AEs in 441 out of 1711 booster recipients, considering factors like age, vaccine brands, and booster combinations. The findings revealed incidence rates (IRs) of 25.6% (95% CI: 21.1–30.8) after the first booster and 24.9% (95% CI: 20.5–30.0) after the second, mostly non-serious, with those having AEs post-first booster being five times more likely to report them again (incidence rate ratio, 5.02, *p* < 0.001). Significantly, switching from the mRNA1273 vaccine to another brand reduced AE risk by 18%. This study underscores that AEs are more repetitive than cumulative with additional booster doses, advocating for personalized vaccination strategies based on individual medical histories and previous vaccine reactions. These insights are valuable for healthcare providers in discussing potential AEs with patients, thereby improving vaccine compliance and public trust, and for policymakers in planning future booster vaccination strategies.

## 1. Introduction

Adverse events (AEs) resulting from repeated COVID-19 vaccine booster doses have become a significant area of research [1,2,3,4]. While vaccines remain one of the most effective means to prevent diseases, vaccine-related adverse events have always been a concern for the public [5,6,7]. Recent studies from large pharmacovigilance databases indicate substantial variability in adverse event incidence rates, emphasizing the need for context-specific analysis in vaccine safety surveillance to maintain public trust and inform future vaccination strategies [8,9,10,11,12]. There are numerous reports of adverse events following COVID-19 vaccination, with particular emphasis on specific groups such as the elderly [13], pregnant women [2,14,15], and those with pre-existing medical conditions. Furthermore, retrospective reports have highlighted vaccine-related neurological adverse events [1,7,16] and cardiovascular complications [3,17,18]. These reports significantly impact public willingness to receive vaccinations, consequently affecting vaccination rates [7,19,20]. Of particular concern are the severe adverse events associated with booster vaccines, such as vaccine-induced myocarditis and pericarditis, multisystem inflammatory syndrome in children (MIS-C), and multisystem inflammatory syndrome in adults (MIS-A) [21,22,23,24,25,26,27]. The question of whether the incidence and severity of side effects increase with additional doses is of utmost importance and urgently requires detailed clinical reports to understand the current situation [28].

Repeated administration of booster doses is crucial for enhancing protective immunity against COVID-19. However, there is a growing need for large-scale studies to support public concerns about the adverse events of these repeated doses. The risk of AEs could be repeated upon subsequent administrations of the same vaccine. Given the variety of COVID-19 vaccines available, each based on different technological platforms such as mRNA and non-replicating viral vectors, potential mechanisms for this could involve immunological responses such as hypersensitivity or immune sensitization, especially relevant in the context of repeated exposures to vaccine antigens [3,13,29]. Moreover, the risk of AEs could be cumulative, potentially due to cumulative immunological effects or other underlying biological factors. This cumulative aspect is paramount in assessing the long-term safety and sustainability of ongoing COVID-19 vaccination strategies, particularly in the context of administering booster doses [30]. A comprehensive analysis of these mechanisms, in terms of pharmacovigilance, is vital for enhancing the efficacy and safety of current and future vaccination strategies against COVID-19 and other diseases.

The primary objective of this study is to investigate the incidence rates of adverse events (AEs) following the administration of the second booster dose (fourth dose) of the COVID-19 vaccine. Taiwan boasts high vaccination rates globally (first dose coverage at 93.8%, second at 89.06%, third at 76.9%, and fourth at 25%) and encourages booster vaccinations for the entire population in contrast to the strategies of other countries that focus primarily on high-risk groups [31,32]. This research utilizes data from the Vaccine Adverse Event Reporting System (VAERS) at Taipei Veterans General Hospital to analyze the types and incidence rates of AEs, and the correlation between the occurrence of AEs after the first and second boosters. Additionally, we evaluate whether the occurrence of AEs is a repeated phenomenon or indicative of a cumulative risk of AEs. Furthermore, we explore the relationship between the choice of vaccine brand and the booster–booster combination in relation to the occurrence of AEs. This comprehensive investigation aims to provide critical insights into the safety profile of repeated booster doses, enhancing the decision-making process for vaccine administration and public health policy.

## 2. Materials and Methods

### 2.1. Taiwan’s Vaccination Program for Booster Vaccines

Taiwan’s booster vaccine program, distinguished by its high vaccination rates (first dose coverage at 93.8%, second at 89.06%, third at 76.9%, and fourth at 25% on 25 December 2023), adopts a comprehensive and inclusive approach. The government’s policy encourages staggered booster vaccinations for the entire population, a contrast to the strategies of other countries that focus primarily on high-risk groups. Integral to this program is a mix-and-match strategy, especially for booster doses, recommending mRNA or subunit vaccines following initial viral vector vaccines to enhance immune response. This strategy adapts to the availability of vaccines, exemplified by the predominant use of the Moderna BA.1 variant vaccine during the fourth dose rollout, due to its availability and the depletion of other brands. The decision for administering the second booster is informed by immunological evidence, allowing for its administration three months after the first booster. A significant focus of the research is the prevalent use of the mRNA1273 (Moderna COVID-19 vaccine, Spikevax (Cambridge, MA, USA)) vaccine in the booster program, particularly notable during the administration of the second booster that included the bivalent version of the Moderna COVID-19 vaccine (vaccine against original strain and BA.4/5 variant, mRNA-1273.214 in Taiwan). This scenario offers a unique opportunity providing insights into the occurrence of adverse events in a real-world context.

### 2.2. Vaccine Adverse Event Reporting System (VAERS) in Taipei Veterans General Hospital

In addition to a centralized Taiwan’s Vaccine Adverse Event Reporting System (VAERS) maintained by Taiwan Centers for Disease Control, Taipei Veterans General Hospital (TVGH) was requested to operate its own VAERS. Individuals who receive their vaccination at TVGH are sent a reminder message seven days post-vaccination, directing them to an online, anonymous questionnaire since most events are reported within a week [33]. They are encouraged to fill out this survey regardless of whether they experienced symptoms or not. This digital survey collects data on the vaccine brand, the dates of both the first and second booster shots, and any post-vaccination symptoms. It includes a section for recipients to describe in their own words the nature of the symptoms (such as fever, fatigue, pain at the injection site, headache, severe allergies, etc.), the timing of their onset after the vaccination, and any follow-up actions they undertook, whether it was self-care at home or seeking outpatient or emergency medical treatment.

A key aspect of this approach is the mandatory reporting of any symptoms by all vaccine recipients, regardless of whether they experience any. This inclusive reporting system guarantees a detailed accumulation of data regarding post-vaccination effects, thereby enhancing the precision and dependability of the study.

### 2.3. Study Design

This cross-sectional study investigated the vaccination status and associated adverse events reported over a three-month period at the end of 2022 at the vaccination site of Taipei Veterans General Hospital. The adverse events were categorized as severe and non-severe by the researchers. The study data included basic demographic information of the participants, vaccination details, and descriptions of any adverse event. The analysis aimed to explore the correlation between vaccine recipients who experienced adverse events and those who did not, examining potential influencing factors.

### 2.4. Data Sources

Data collection spanned from the announcement of the second booster (fourth dose)’s availability to citizens and foreign residents over 18 years old on 27 October 2022 until 19 January 2023. Patients receiving vaccines underwent thorough medical examinations, including checks for allergic reactions, acute infection symptoms, and major vaccine-related adverse events. Post-vaccination, patients were provided with a Chinese-language Google Form questionnaire, accessible via a QR code, to report any adverse events within seven days of vaccination. The questionnaire included demographics, vaccine brand choice for the second booster, and any adverse event experienced. To ensure privacy, gender was not included in the questionnaire. The information was anonymized upon submission, with each respondent limited to one submission. This study, approved by the IRB of Taipei Veterans General Hospital (IRB 2022-12-005AC#1), ensured comprehensive data collection while maintaining participant confidentiality.

### 2.5. Study Population

**Figure 1:**
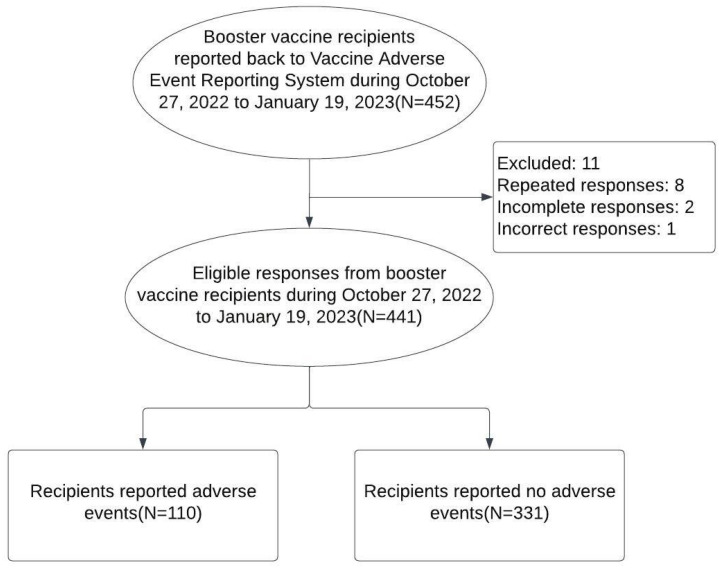
Study flow of participant enrollment and outcomes for adverse events (AEs) reporting following the second COVID-19 booster at Taipei Veterans General Hospital (27 October 2022–19 January 2023).

The study population comprised individuals who sent their response after receiving vaccinations at the vaccination site of Taipei Veterans General Hospital between 27 October 2022 and 19 January 2023. Responders with duplicate submissions, incomplete responses, and incorrect responses were excluded (Figure 1).

### 2.6. Basic Demographic Information and Vaccination History

Basic information, including respondents’ age and vaccination history, including the brand and time of each dose of vaccine, were extracted from the VAERS. First boosters and second boosters with the same brand of boosters were considered as homologous, and were otherwise classified as heterologous. Vaccines were further divided into two groups of “mRNA1273 (Moderna)” and “others” because of the high share of mRNA1273 vaccines.

### 2.7. Classification of AEs, Serious Adverse Events (SAEs), and Non-Serious Adverse Events (NSAEs)

Adverse event reports were self-submitted by participants with settled options in the questionnaire and also their own descriptions. Regardless of primary doses, only AEs after the first booster were considered. The responses were categorized by Lin, C.H. and Chen, Y.C. based on the questionnaire’s design. Symptoms potentially related to the cardiovascular system (like chest tightness or difficulty breathing) were classified as Serious Adverse Events (SAEs). Other symptoms, including localized reactions at the injection site, flu-like symptoms, rapid heartbeat, gastrointestinal issues, and muscle aches, were categorized as Non-Serious Adverse Events (NSAEs). This classification was utilized to analyze the correlations and potential influencing factors between vaccine recipients who experienced adverse events (both SAEs and NSAEs) and those who did not report any adverse events.

The first author (Lin, C.H.) classified the multiple descriptions into 6 domains: “cardiac symptoms”, “local reactions”, “flu-like symptoms”, “gastrointestinal symptoms”, “muscle/joint pain”, and “others” as described in another previous study [34]. AEs reported after the first and second boosters were referred to as first and second AEs, respectively.

### 2.8. Definition of Repeated Adverse Events

Repeated adverse events (repeated AEs) refers to the occurrence of adverse events following each subsequent dose of the COVID-19 vaccine. This category is particularly focused on analyzing whether individuals who experienced AEs after one dose are more likely to experience similar events following subsequent doses. Cumulative risk of AEs (cumulative AEs) assesses whether the likelihood or severity of adverse events increases with each subsequent dose of the vaccine. This concept is crucial for evaluating the long-term safety of the vaccination, especially in a regime involving multiple booster doses. In the current study, we investigated incidence-dependent cumulative risk by evaluating whether the incidence of AEs increases with additional doses.

### 2.9. Statistical Analysis

To estimate the incidence rates of AEs, we employed point estimation using a binomial distribution to calculate the incidence rates (IRs) and 95% confidence interval (95% CI) of AEs for each category. We used the Fisher exact test to compare the effects of various factors and identify the factors associated with the occurrence of AEs after the second booster. Furthermore, Poisson regression analysis was used to identify factors associated with AEs following the second booster. Incidence rate ratios (IRRs) were used to evaluate the effect of association. To test repeated occurrences of AEs, the McNemar test was used to explore the association between first AEs and second AEs. We examined the IRs of first AEs and second AEs to evaluate the cumulative risk of AEs. All statistics were analyzed using R (R-4.3.2 for Windows); a *p*-value < 0.05 was considered statistically significant.

## 3. Results

### 3.1. Population Characteristics and AE Reports from VAERS

Over a three-month period, Taipei Veterans General Hospital administered COVID-19 vaccinations to 1711 individuals. From this group, 452 reported their post-vaccination outcomes to VAERS between 27 October 2022 and 19 January 2023. A total of 441 respondents were ultimately included in this analysis. Of these, 113 reported adverse events (first AEs) following their first booster and 110 reported adverse events (second AEs) after the second booster. This resulted in incidence rates (IRs) of 25.6% (95% CI: 21.1–30.8) after the first booster and 24.9% (95% CI: 20.5–30.0) after the second. Notably, AEs occurred repeatedly for individuals with first AEs; a McNemar test showed similar IRs for either first AEs or second AEs (difference −0.68%, 95% CI, −4.63% to 3.27%, *p*-value = 0.8221) (Table 1).

The incidence of second AEs appears to be influenced by age, with younger recipients (ages 18–39: 27.8%; 40–64: 24.7%) experiencing a higher rate of AEs compared to those aged 65 and above (14.6%). This trend suggests a greater occurrence of AEs among younger age groups. Additionally, the IRs of second AEs showed variation based on the vaccine brand, with mRNA-1273 having 100 cases and an IR of 26% (95% CI: 21.7–30.4). However, it is important to note that no statistically significant differences were found concerning age and vaccine brand, indicating that these factors did not significantly affect the incidence of AEs in this study (Table 1).

### 3.2. Analysis of Second AEs Reported by Recipients of the COVID-19 Second Booster Vaccines

The majority of adverse events (AEs) reported following the second COVID-19 booster vaccination were classified as Non-Serious Adverse Events (NSAEs). The incidence rate (IR) of Serious Adverse Events (SAEs) for the second booster was relatively low, at 1.3% (6 cases, 95% CI: 0.5–3.0). Among the NSAEs, the most common were pain at the injection site (97 cases, IR: 22%), fatigue (68 cases, IR: 15.4%), headache (60 cases, IR: 13.6%), and fever (41 cases, IR: 9.3%). The concurrent occurrence of pain at the injection site, fatigue, and headache was notably frequent, observed in 18 cases (IR: 16%). Additionally, the data suggest a lower IR of second AEs for brands other than mRNA1273, with an IR of 17.5% compared to 26.0% for mRNA1273 across all types of AEs (Table 2). Responders of younger age were more likely to report AEs after their boosters (Supplement Figure S1).

### 3.3. Influential Factors of Second AEs

**Figure 2:**
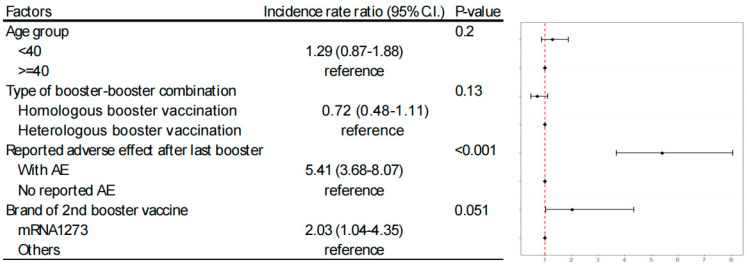
Influential factors for adverse event (AE) reporting following the second COVID-19 booster at Taipei Veterans General Hospital (2022 October 2–19 January 2023).

Using Poisson regression analysis, we evaluated factors such as age, type of booster–booster combination, and the occurrence of first adverse events (AEs). The analysis identified that the occurrence of first AEs was the most significant predictor (incidence rate ratio, 5.41, *p* < 0.001) for second AEs. Individuals who had adverse events with an mRNA vaccine as their first booster were more prone to similar reactions with the same vaccine type for their second booster. Although the influence of vaccine brand choice did not reach statistical significance (incidence rate ratio, 2.03, *p* = 0.051), there was a trend indicating that participants receiving mRNA1273 had a higher incidence rate of adverse events (Figure 2).

**Figure 3:**
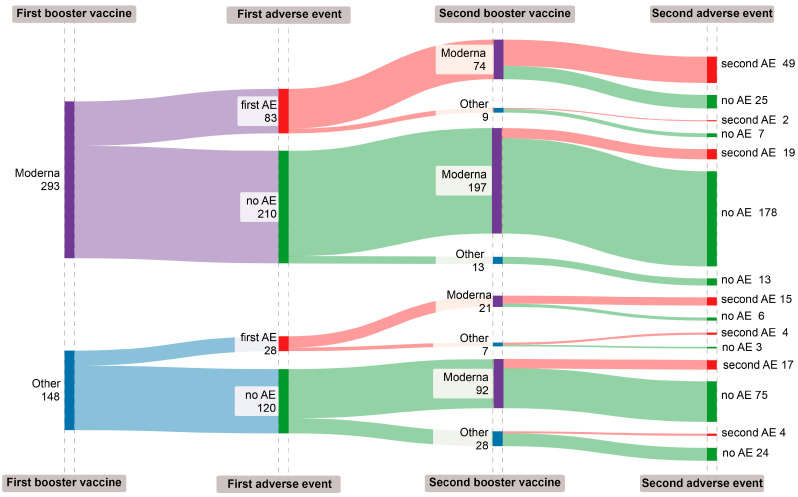
Sankey diagram of occurrence of adverse events (AEs) reported following the first and second COVID-19 boosters at Taipei Veterans General Hospital from 27 October 2022 to 19 January 2023. Dark purple blocks denote receipt of mRNA-1273; dark blue blocks denote receipt of other brands of vaccine; dark red blocks denote occurrence of adverse events (first AE or second AE); dark green blocks denote no adverse events (no AEs); the following number denotes the number of recipients (n = 441).

The incidence of adverse events (AEs) following COVID-19 booster vaccinations appears to be repetitive, yet the cumulative risks associated with these AEs were complex (Table 3, Figure 3). Individuals who received mRNA1273 for both doses had a stable AE rate, with 26.0% after the first booster (95% CI: 21.2–31.7) and a minor decrease to 25.1% post-second booster (95% CI: 19.5–31.8). Those who had mRNA1273 followed by a different brand observed a notable reduction in AEs to 9.1% after the second booster (95% CI: 1.1–32.8). In contrast, participants initially given a different brand, then mRNA1273, experienced an increase in AE incidence from 17.5% after the first booster (95% CI: 8.4–32.3) to a higher rate after the second mRNA1273 booster compared to those who continued with a non-mRNA1273 brand (28.3% vs. 21.6%, respectively). This suggests that for those who experienced adverse events with an mRNA1273 vaccine during their first booster, choosing a different vaccine brand for the second booster might lower the risk of subsequent adverse events (Table 3).

## 4. Discussion

To address widespread public concerns about adverse events (AEs) due to repeated vaccinations, this study analyzed VAERS data from Taipei Veterans General Hospital to explore the AEs following the administration of the second COVID-19 booster dose. The incidence rates of adverse events (AEs) were relatively high, at 25.6% after the first booster and 24.9% after the second. Most notably, these AEs were predominantly non-serious, with symptoms such as injection site pain and fatigue being the most common. We observed the pattern of adverse events (AEs) following COVID-19 booster vaccinations to be repetitive rather than cumulative, where individuals who experienced an AE following the first booster were more likely to report AEs after the second booster. This finding underscores the importance of considering previous AEs, the brand of the second booster, and the booster combination in devising personalized vaccination strategies. These results are vital for health providers and enhancing the understanding of vaccine safety, particularly in the context of administering booster doses.

This study offers crucial insights into the occurrence of adverse events (AEs) following COVID-19 booster vaccinations, particularly in a real-world setting in Taiwan where vaccinations are not limited to priority groups. In our study, we observed a considerable incidence rate of adverse events (AEs) post COVID-19 booster vaccinations. It is essential to recognize that the majority of these AEs were minor, frequently manifesting as injection site pain, fatigue, or low-grade fever. The finding is compatible with a recent systematic review of 22 randomized controlled trials involving over 3.4 million older adults reporting an overall incidence rate of adverse events at 36.6% [4]. The temporal association of these AEs following vaccination does not necessarily imply causation, and it is important to consider the possibility of temporal bias. Additionally, the reliance on self-reported data may lead to an inflated incidence rate due to the underreporting of non-events. There is notable variability in AE incidence rates across large pharmacovigilance databases, as seen in recent studies [8,9,10,11,12]. Our findings contribute further insights into common but non-fatal AEs, consistent with the general profile of vaccine-related side effects that are typically transient and resolve without serious consequences [2,8,9,10,11,35,36]. Therefore, the high incidence rate observed should be interpreted within the larger framework of vaccine safety, emphasizing that the benefits of protection against COVID-19 far outweigh the minor discomforts associated with these non-serious AEs, a fact that should be clearly communicated to address public concerns.

Our observations indicate that individuals who experienced side effects after their first COVID-19 booster often faced similar issues with subsequent doses. Conversely, those who did not encounter side effects initially tended to remain symptom-free after additional doses. This finding is compatible with findings in a study focusing on AEs after primary series in Japan [37]. This trend may be linked to the body developing a heightened sensitivity or allergic response, particularly in those already predisposed to such reactions. Ingredients in the vaccines, such as polyethylene glycol (PEG) and lipid nanoparticles (LNPs), could be responsible for triggering these responses in sensitive individuals [30]. The presence of vaccine adjuvants, which boost the body’s response to the vaccine, could also play a role. Understanding why these reactions happen is important for finding ways to prevent side effects in people who are more likely to have them, especially when considering their past experiences with allergies and sensitivities during booster vaccinations. Additionally, it is important to consider the possibility of reporting bias, where individuals more conscious of their adverse events (AEs) might be more likely to report them, potentially influencing our findings.

Our study found no evidence indicating that adverse events (AEs) increase in frequency with successive COVID-19 booster doses within the same demographic group. This suggests that although an effective immune response is crucial for vaccine effectiveness, and repeated vaccinations may activate the immune system, this does not necessarily lead to a higher frequency of adverse events (AEs) with each subsequent booster dose. This insight is particularly relevant considering the World Health Organization’s (WHO) updated guidelines in 2023, which now recommend booster vaccinations mainly for priority groups, following a comprehensive assessment of the risks and benefits associated with additional doses. [38,39]. Our results provide reassuring evidence regarding the safety of administering second booster doses in public COVID-19 vaccination programs. This information is essential for public health communication, offering reassurance about the safety of receiving multiple booster doses during ongoing efforts to combat COVID-19.

Cautions are needed for interpretation the current result. The reliance on self-reported data in our research might have led to an overestimation of the incidence rates of adverse events. This is particularly relevant as self-reported data can vary in accuracy and completeness, which may skew the true incidence of these events. It is important to note that our findings predominantly apply to common and minor adverse events, given that the number of cases reported was relatively small. Therefore, these results may not be generalizable to rare and severe adverse events. This limitation underscores the need for further studies with more robust data collection methods to accurately assess the incidence of both common and rare adverse events following COVID-19 booster vaccinations.

The association between comorbidities and occurrence of adverse events remained unanswered, while some observational studies noting a higher percentage of adverse events among individuals with comorbidities [12]. Contrarily, some study found no significant increase in risk or severity of vaccine-related side effects in participants with comorbidities [40]. However, our study did not include data on participants’ comorbidities or prior COVID-19 infections, limiting our ability to analyze these factors’ impact on vaccine response and adverse events. This highlights the need for further research on a larger scale specifically focused on individuals with multiple comorbidities to better understand the risks associated with COVID-19 vaccination in this population.

In our study, we limited the reporting of adverse events to within 7 days post-vaccination to increase response rate and minimize recall bias. This approach, however, may have introduced bias by potentially overlooking adverse events that occur weeks later and generally capturing only common and minor reactions. It is noteworthy that a study revealed the median time to embolic event onset was 6 days for mRNA vaccines and 11 days for viral vector vaccines (those were not used as a booster), partially aligning with our study’s reporting period [33]. Therefore, the interpretation of our study’s results should be warranted, considering these limitations and the generally captured common and minor reactions in the short term.

Moreover, there are still some limitations to the current study. First, a significant limitation of this study is reporting bias. The reliance on self-reported data means patients without adverse events may be less inclined to report their status, potentially leading to an overestimation of the incidence of adverse events. This bias can skew the results towards those who experienced more noticeable or bothersome symptoms. Moreover, a significant limitation of this study is the absence of gender data in the questionnaire. The lack of gender-specific information precludes analysis of gender differences in vaccine responses, this gap in data may impact the study’s relevance to diverse populations. Secondly, the study’s methodology is susceptible to recall bias. Since data collection is based on participants’ recollections of their symptoms and the timeline of these symptoms post-vaccination, participants may forget minor symptoms or misremember the severity and timing of their symptoms. Third, the intensity of discomfort or severity of adverse events in this study was subjectively evaluated by the participants themselves, rather than through objective assessment by medical experts. This subjective evaluation can lead to a variation in the reported intensity of symptoms. Fourth, the study’s findings may not be generalizable to the broader population due to the limited sample size and the specific demographic characteristics of the study participants. Larger and more diverse sample sizes are needed to validate these findings across different populations. Fifth, the study does not account for all possible confounding factors, such as underlying health conditions, concurrent medication use, or previous exposure to COVID-19, which could influence the occurrence and reporting of adverse events. Sixth, the reliance on online questionnaires for data collection may introduce a selection bias, as it excludes individuals without internet access or those who are less technologically inclined. Seventh, the study establishes a temporal association between vaccination and the occurrence of adverse events but does not prove causality. Further investigation is needed to establish a direct causal relationship. Furthermore, our study did not include data on participants’ prior infections with COVID-19, nor did it encompass information regarding their comorbidities, limiting our ability to analyze the impact of previous COVID-19 infections and existing health conditions on vaccine response and adverse events.

## 5. Conclusions

Our study indicates that approximately one-quarter of individuals receiving COVID-19 booster vaccines reported adverse events (AEs), predominantly of a non-serious nature. Notably, these AEs tend to recur in individuals with previous adverse reactions rather than accumulate over successive doses. This repetitive nature of AEs highlights the importance of considering individual medical histories in vaccination plans. These findings are crucial for shaping personalized vaccination strategies, particularly for those with a history of AEs. The insights gained may aid healthcare providers in informing patients about potential AEs, enhancing vaccine compliance and public trust.

## Figures and Tables

**Table 1:** Occurrence of adverse events among respondents receiving the second booster at Taipei Veterans General Hospital vaccination site between 27 October 2022 and 19 January 2023 (n = 441).

	No. of Respondents		Occurrence of Any Adverse Event after Second Booster		
Factors	Count	(%)	Count	Incidence Rate per 100 Respondents (95% CI)	*p*-Value ^1^
Overall	441	(100.0)	110	24.9 (20.9–29.0)	
Age group					0.215
<40	169	(38.3)	47	27.8 (21.1–34.6)	
<65	231	(52.4)	57	24.7 (19.1–30.2)	
>=65	41	(9.3)	6	14.6 (3.8–25.5)	
Type of booster–booster combination					0.983
Homologous booster vaccination	279	(63.3)	69	24.7 (19.7–29.8)	
Heterologous booster vaccination	162	(36.7)	41	25.3 (18.6–32)	
Brand of first booster					0.547
mRNA1273	293	(66.4)	70	23.9 (19.0–28.8)	
Others	148	(33.6)	40	27.0 (19.9–34.2)	
Occurrence of adverse events (AEs) after first booster					<0.001
First AE reported	113	(25.6)	72	63.7 (49.9–80.2)	
No first AE	328	(74.4)	38	11.6 (8.1–15.0)	
Brand of second booster					0.223
mRNA1273	384	(87.1)	100	26.0 (21.7–30.4)	
Others	57	(12.9)	10	17.5 (7.7–27.4)	

^1^—Fisher exact test.

**Table 2:** Occurrence of adverse events by second booster brands among respondents receiving their second booster at Taipei Veterans General Hospital vaccination site during the first wave of the public vaccination program (n = 441).

	Overall (n = 441)		mRNA1273 (n = 384)		Other (n = 57)		
	Count ^1^	IR ^2^ (%)	Count ^1^	IR ^2^ (%)	Count ^1^	IR ^2^ (%)	*p*-Value ^3^
Total no. of any adverse events	110	24.9	100	26.0	10	17.5	0.223
Serious Adverse Events (SAEs)							
Cardiac symptoms							
Chest pain	4	0.9	4	1.0	0	0	1.000
Short of breath	3	0.7	2	0.5	1	1.8	0.341
Non-Serious Adverse Events (NSAEs)							
Local reactions	97	22	89	23.2	8	14.0	0.167
Flu-like symptoms							
Tiredness	68	15.4	61	15.9	7	12.3	0.612
Headache	60	13.6	57	14.8	3	5.3	0.078
Fever	41	9.3	40	10.4	1	1.8	0.063
Chillness	4	0.9	4	1.0	0	0	1.000
Palpitation	3	0.7	2	0.5	1	1.8	0.341
Gastrointestinal symptoms							
Nausea	3	0.7	3	0.8	0	0	1.000
Muscle/joint pain	9	2.0	7	1.8	2	3.5	0.328
Others	7	1.6	7	1.8	0	0	1.000

^1^—The total number exceed 110 since one may have more than one adverse events; ^2^—Incidence rate; ^3^—Fisher exact test.

**Table 3:** Incidence rates of adverse events by brands of first and second boosters among respondents receiving their second booster at Taipei Veterans General Hospital vaccination site during the first wave of the public vaccination program (n = 441).

		Incidence Rate of Adverse Event after First Booster (First AE)		Incidence Rate of Adverse Event after Second Booster (Second AE)	
Brand of First Booster	Brand of Second Booster	Incidence Rate (%)	95% Confidence Interval	Incidence Rate (%)	95% Confidence Interval
mRNA1273	mRNA1273	26.0	(21.2–31.7)	25.1	(19.5–31.8)
mRNA1273	Other	26.0	(21.2–31.7)	9.1	(1.1–32.8)
Other	mRNA1273	17.5	(8.4–32.3)	28.3	(19.4–40.0)
Other	Other	17.5	(8.4–32.3)	21.6	(9.3–42.6)

## Data Availability

The original data are unavailable due to privacy or ethical restrictions.

## Supplementary Materials

The following supporting information can be downloaded at: https://www.mdpi.com/article/10.3390/vaccines12020149/s1, Figure S1: Occurrence and proportions of adverse events after second booster by age of respondents receiving their second booster at Taipei Veterans General Hospital vaccination site during the first wave of the public vaccination program (n = 441).
