# Supplementary figures and images for: Incidence and Nature of Short-Term Adverse Events following COVID-19 Second Boosters: Insights from Taiwan’s Universal Vaccination Strategy
Source: Vaccines (Basel). 2024 Jan 31;12(2):149. doi: 10.3390/vaccines12020149 (PMC10892656; doi:10.3390/vaccines12020149)

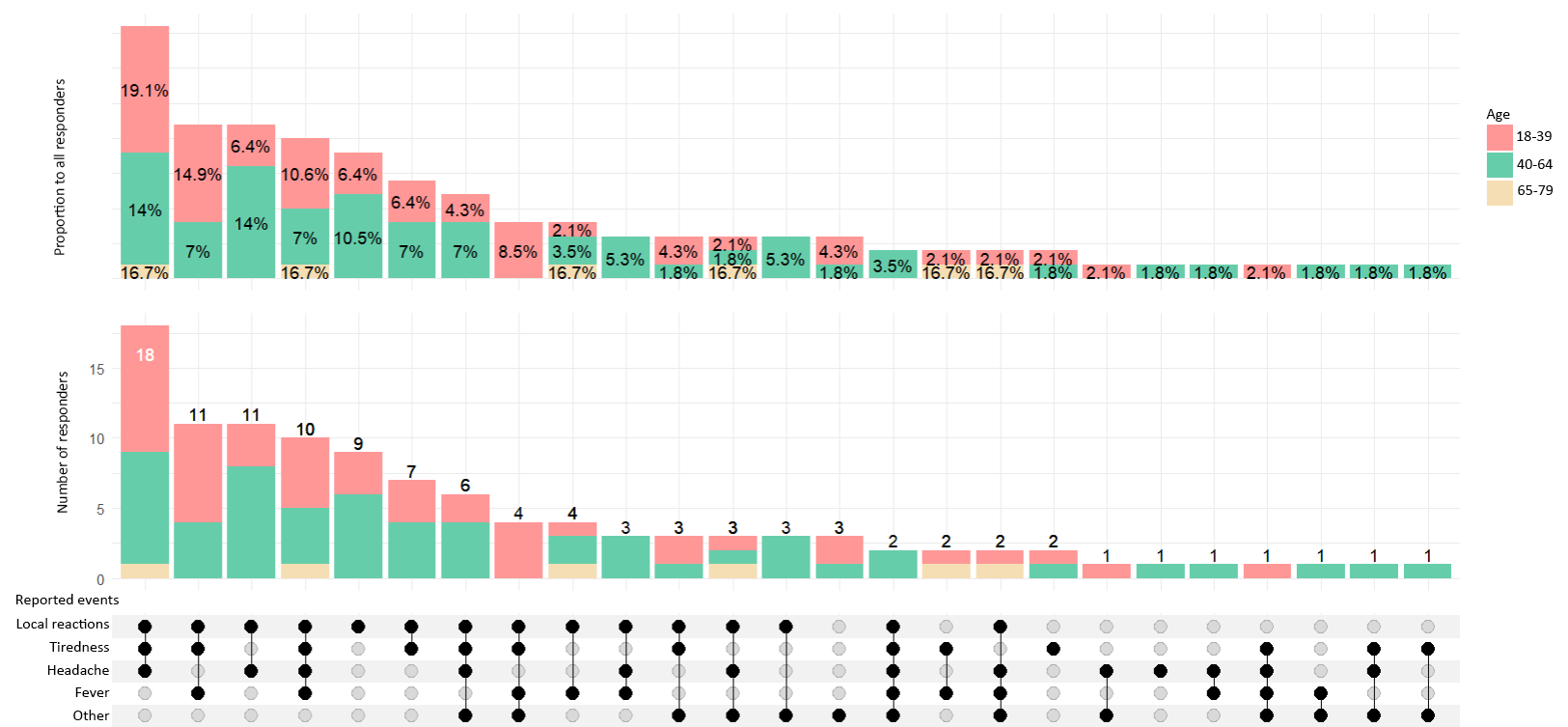

Supplement: Supplementary file 1 [file vaccines-12-00149-s001.zip › vaccines-2829491-supplementary.png]
